# An Efficient Uplink 3D AoA Positioning Framework for 5G RedCap UEs in Indoor Factory Environments

**DOI:** 10.3390/s26134176

**Published:** 2026-07-02

**Authors:** Ilya Averin, Andrey Pudeev, Seunggye Hwang, Hyunsoo Ko

**Affiliations:** 1 Russia R&D Lab, LG Electronics Inc., 28, Bolshoy Sampsoniyevskiy Avenue, Bldg. 2, 195277 Saint Petersburg, Russia; 2 LG Electronics Inc., 19, Yangjae-daero 11-gil, Seocho-gu, Seoul 06772, Republic of Korea

**Keywords:** indoor positioning, 5G RedCap, angle of arrival (AoA), antenna arrays, weighted least squares (WLS), spatial covariance matrix, multipath mitigation, Indoor Factory (InF), Industrial Internet of Things (IIoT)

## Abstract

This paper addresses the challenge of Reduced Capability (RedCap) User Equipment (UE) positioning within indoor 5G networks. While conventional approaches rely on time-domain ranging, the limited signal bandwidth associated with RedCap devices compromises the capability of these methods to satisfy stringent accuracy requirements. To overcome this limitation, we propose a positioning framework based on uplink Angle-of-Arrival (AoA) measurements. By performing AoA estimation at the Transmission and Reception Point (TRP), the proposed approach maintains hardware simplicity, requiring only a single antenna at the UE. The framework incorporates a computationally efficient AoA estimation algorithm derived from the analysis of the spatial covariance matrix, eliminating the need for the exhaustive beam scanning typically required for angular grid search. This procedure inherently generates a link quality metric which, alongside the AoA estimate, is utilized for final UE localization. The localization algorithm employs a Weighted Least Squares (WLS) estimator to provide a unified approach to UE positioning in both 2D and 3D physical spaces. The framework’s efficacy is confirmed via numerical simulations under the dense multipath conditions defined by standard 5G Indoor Factory (InF) environments.

## 1. Introduction

The ongoing evolution of fifth-generation (5G) networks and the subsequent transition to sixth-generation (6G) networks are driven by rising commercial and technical demands. The diverse set of 5G applications is characterized by the well-known triangle formed by Enhanced Mobile Broadband (eMBB), Ultra-Reliable Low Latency Communications (URLLC), and Massive Machine-Type Communications (mMTC). While the primary objective within each group of applications is to maximize communication performance, the capability to perform positioning is of crucial importance for many existing and potential applications.

In the context of 5G communication systems, positioning is a procedure aimed at estimating the unknown coordinates of user equipment (UE), under the assumption that the coordinates of the transmission and reception points (TRPs) are known. The TRPs involved in this process are commonly referred to as reference nodes or anchor points.

A variety of ambitious 5G applications, such as autonomous vehicles, digital twins, and augmented reality, can potentially benefit from high-accuracy positioning. Furthermore, access to location-aware services is of great practical interest for a range of industrial segments known as Industry 4.0. For example, highly automated plants may require high-precision positioning for gathering information about resource placement, as well as for tracking assets and tools in both production and storage. As 5G deployments gain momentum, the challenge of 5G positioning and, specifically, indoor positioning, is attracting significant research interest [1,2].

The performance of widespread time-domain positioning methods based on Time of Arrival (ToA) or Time Difference of Arrival (TDoA) estimation is known to be strongly dependent on the signal bandwidth (BW) [2]. While a wide signal BW is inherent to some 5G use cases, this is not always the case. In particular, 3GPP Rel-18 features the positioning of Reduced Capability (RedCap) UEs [3]. RedCap UEs include wearables (e.g., smartwatches, medical devices, and AR/VR goggles), industrial wireless sensors and video surveillance.

The primary design objective of mid-tier RedCap UEs is to minimize hardware cost and complexity compared to high-end eMBB and URLLC devices. This requires significant hardware simplification and, more importantly for positioning, a reduction in signal BW. Specifically, the maximum UE bandwidth is 20 MHz for FR1 and 100 MHz for FR2. At the same time, stringent positioning performance must be maintained. For commercial use cases, the horizontal and vertical positioning accuracy should be better than 3 m, while for industrial use cases, the target is under 1 m (horizontal) and 3 m (vertical) for 90% of UEs [4]. Recent systematic surveys of the 5G RedCap landscape [5] highlight that while high-complexity techniques like carrier phase positioning offer centimeter-level accuracy, they pose significant implementation challenges for resource-constrained devices. This necessitates a search for more practical, network-native alternatives suitable for bridging the RedCap performance gaps.

Given the BW limitations, achieving the required positioning accuracy with ToA/TDoA approaches may be challenging. Therefore, alternative methods must be considered. One such approach is based on Angle of Arrival (AoA) measurements [1]. The primary advantage of angular-domain positioning is that its performance is only weakly dependent on the signal BW [6]. A practical drawback of AoA-based methods is the requirement for multiple-antenna systems at the receiver. However, antenna arrays are already widely used for communication and are an inherent part of 5G networks. Furthermore, AoA estimation can be performed on the uplink. Consequently, AoA-based positioning can be implemented for RedCap UEs equipped with only a single antenna.

In multipath conditions, the accuracy of AoA estimation is known to degrade more severely than that of ToA/TDoA [6]. This can lead to a significant reduction in positioning performance. Since dense multipath environments are inherent to 5G indoor deployments (e.g., the Indoor Factory (InF) scenario [7]), obtaining accurate AoA estimates for RedCap UEs may be challenging. Nevertheless, the high density of TRPs ensures that multiple AoA estimates are potentially available. Consequently, rather than focusing solely on the precision of individual estimates, one can utilize a link quality metric as an indicator of estimation quality. This system-level approach aligns with recent experimental findings from open-source 5G testbeds [8], which emphasize the importance of phase calibration and uncertainty management in real-world uplink AoA implementations.

The core scientific contributions and novelty of this work are centered on a unified estimation and weighting architecture that bridges physical-layer spatial channel behavior with system-level geometric localization. Unlike traditional positioning pipelines where AoA estimation and link-reliability assessment are treated as independent, disjointed tasks, the proposed framework extracts a link quality metric as a direct, zero-overhead byproduct of a linear phase regression over the antenna array’s principal eigenvector. By mathematically demonstrating that these phase-fitting residuals serve as a physical proxy for the angular estimation variance, a rigorous Gauss–Markov justification for the mapping of physical wavefront distortions into a 3D block-diagonal weighting matrix is established. This joint coupling between physical-layer wavefront planarity and overdetermined projective geometry eliminates the risk of geometric degradation common to hard-thresholding techniques, introducing an autonomous “soft-filtering” mechanism that aims to preserve system stability in harsh industrial topologies.

The main contributions of this paper are as follows:

- Search-free AoA Estimation: A computationally lean algorithm for 1D and 2D Angle-of-Arrival (AoA) estimation leveraging the principal eigenvector of the spatial covariance matrix. This approach eliminates the need for multi-dimensional grid searches and applies to both Uniform Linear Arrays (ULAs) and Uniform Rectangular Arrays (URAs). By shifting the spatial processing to the uplink, the framework enables AoA-based positioning while maintaining a strict single-antenna configuration at the RedCap UE.
- Intrinsic Link Quality Metric: A novel metric generated inherently during the AoA estimation process. It establishes an embedded, mathematical link between wavefront phase residuals and physical estimation variance, transforming wavefront distortion into a predictable proxy for angular uncertainty.
- Unified Localization Engine: A 3D UE localization algorithm utilizing the derived AoA estimates and link quality metrics within a Weighted Least Squares (WLS) framework. It provides a consistent mathematical approach for positioning across both 2D and 3D physical spaces, leveraging an autonomous “soft-filtering” mechanism to suppress AoA estimation outliers without degrading network geometry.

The potential effectiveness of the proposed positioning framework in the dense multipath conditions inherent to 5G indoor environments is evaluated via the presented simulation results. Under the chosen high-fidelity simulation settings, the findings validate the theoretical feasibility of meeting stringent positioning accuracy requirements for RedCap UEs even in the challenging InF-SH scenario, establishing a foundation for future physical deployment validation.

## 2. Positioning Framework

### 2.1. Related Works

#### 2.1.1. Time-/Power-Domain vs. Angular-Domain Positioning

The most common approach to 5G positioning is based on time-domain ranging [9]. The underlying principle of the ToA method is to measure the propagation delay between transmission and reception, which is subsequently translated into the distance between a transmitter (e.g., the UE) and a receiver (e.g., the TRP). For ToA-based ranging to be effective, the TRP and the UE must be strictly synchronized. This requirement can be relaxed by transitioning to the TDoA method, which utilizes the difference in arrival delays between the UE and two or more TRPs. In this scenario, only the TRPs must be synchronized, which simplifies the practical implementation of the positioning system.

Both ToA and TDoA methods can potentially provide high ranging accuracy, even in the dense multipath conditions inherent to 5G indoor deployments. This accuracy can be further enhanced by utilizing carrier phase (CP) measurements [10]. However, to achieve the extremely high precision offered by CP-based methods, the integer ambiguity problem must be resolved. In general, this requires additional system resources, such as multiple carriers or a greater number of TRPs compared to standard ToA/TDoA approaches.

Once the time delays corresponding to several unique TRP-UE pairs are available, the UE coordinates can be determined by solving a trilateration (or multilateration) problem. This problem has a clear geometric interpretation [11]: for ToA, each delay value defines a sphere centered on the corresponding TRP, while for TDoA, each pair of delay values defines a hyperboloid with foci at the respective TRPs. Ideally, if measurements are error-free, the UE coordinates are located at the intersection of these spheres or hyperboloids. Consequently, trilateration is a fundamentally non-linear problem. While some linearization techniques are possible [11], solving the original non-linear problem can require considerable computational resources.

It is straightforward to see that any timing jitter between reference nodes translates directly into a dilution of precision for TDoA-based positioning. Nevertheless, in the context of 5G networks, the required nanosecond-level time synchronization can be maintained.

The fundamental operational limits of these conventional frameworks can be categorized into distinct physical bottlenecks when applied to mid-tier industrial infrastructure:

- ToA/TDoA Bottleneck: Ranging precision is fundamentally bounded by signal bandwidth [2]. 3GPP Rel-18 RedCap UEs are strictly limited to a narrow 20 MHz maximum bandwidth in FR1. At 20 MHz, the basic sampling period is 50 nanoseconds, translating to a coarse geometric resolution of roughly 15 m per sample. Furthermore, multipath interference is more problematic for narrowband signals, as the signal’s temporal characteristics become wide relative to the channel delay spread [6]. Resolving dense, sub-meter industrial multipath in the time domain under these narrowband constraints is highly challenging and computationally demanding.
- RSSI/Power Bottleneck: While RSSI methods offer low hardware complexity, the heavy metallic structures and moving machinery inherent to an InF environment induce severe shadowing and dynamic path attenuation. This completely decouples received signal power from physical distance, resulting in massive localization bias unless complex, fragile fingerprinting databases are continuously maintained [12]. It is worth noting that fingerprinting requires an extensive offline calibration stage and lacks robustness against environmental changes or setup alterations [1].
- AoA Advantage: In contrast, angular-domain positioning accuracy relies on the spatial aperture (number of antenna elements) at the receiver, making its estimation performance weakly dependent on the signal bandwidth. This makes uplink AoA the ideal alternative for bridging the RedCap performance gaps.

Consequently, AoA-based positioning emerges as a highly viable approach for high-precision RedCap applications, although its effectiveness depends heavily on the specific estimation strategy and its resilience to multipath interference, as discussed in the following sections.

#### 2.1.2. AoA Estimation Strategies and Their Limitations

Angle-based positioning utilizes information regarding the direction from which a signal is received, specifically through AoA measurements. The primary advantage of AoA-based positioning is that estimation accuracy is largely independent of the signal BW. Consequently, AoA-based methods can perform effectively with narrowband or even unmodulated signals [6]. This imposes significantly more relaxed bandwidth requirements compared to ToA, TDoA, and CP techniques. Moreover, AoA-based positioning has less stringent time synchronization requirements than those of time-domain methods [6].

Provided that the AoAs corresponding to several unique TRP-UE pairs are available, the UE coordinates can be determined by solving a triangulation problem. Similar to trilateration, triangulation has a clear geometric interpretation [11]: assuming error-free measurements, the UE coordinates are located at the intersection of rays originating from the respective TRPs. Consequently, unlike trilateration, triangulation can be formulated as a fundamentally linear problem. Furthermore, only two reference points are required to perform AoA-based positioning in 3D space, which offers a significant practical advantage over ToA/TDoA-based methods that require at least four. Accordingly, AoA-based techniques represent a promising alternative for the positioning of RedCap UEs.

Despite its advantages, AoA-based positioning possesses certain limitations. First, a practical drawback is the requirement for specialized hardware to perform AoA estimation. While the field of AoA estimation has been established in radar and other sensing systems for decades, it generally relies on phase-based methods [13]. This approach evaluates the orientation of the constant-phase wavefront and can be implemented in various ways. For example, AoA can be estimated by rotating a directional antenna; in this case, the AoA estimate corresponds to the direction where the RSS is maximized or minimized.

With advancements in digital signal processing, another phase-based approach known as spatial aperture sampling [13] has seen widespread adoption. While this can take various forms, it is most commonly implemented using antenna arrays. To ensure accurate AoA estimation, these arrays must be calibrated following installation and periodically maintained to minimize measurement uncertainty. Nevertheless, given that antenna arrays are already extensively utilized in 5G systems for communication, their reuse for positioning provides a significant added benefit. Furthermore, by performing positioning on the uplink, all complex processing and hardware requirements are shifted to the TRPs. This allows the hardware of RedCap UEs to remain simple, requiring only a single antenna.

A more fundamental drawback of AoA-based positioning is that its accuracy is determined by both the precision of the AoA estimate and the distance between the TRP and the UE. This stands in contrast to ToA/TDoA methods, where ranging accuracy depends primarily on the precision of the delay measurements. While this distance dependency can be a limiting factor in outdoor positioning, the indoor environment typically involves much shorter TRP-to-UE distances. Consequently, provided the AoA estimation is sufficiently accurate, high positioning precision can be achieved. This suggests that the bottleneck for such systems lies primarily in the accuracy of the AoA estimation itself.

Numerous AoA estimation methods for antenna arrays have been presented in the literature. The beamformer developed by Bartlett [14] is widely considered a reference technique, known for its effectiveness and robustness against hardware impairments. However, its performance can significantly degrade in the dense multipath conditions inherent to indoor deployments. This degradation is primarily caused by wavefront distortion, which occurs when multiple replicas of the transmitted signal arrive at the receiver from closely spaced angles.

To address this challenge, one may apply super-resolution techniques, such as Capon’s algorithm [15] or subspace-based methods like MUSIC [16] and ESPRIT [17]. Other notable techniques include the Maximum Likelihood (ML) estimator [18] and the computationally efficient Root-MUSIC algorithm [19]. While these methods can provide superior AoA estimation accuracy, they often require significant computational resources. Furthermore, super-resolution algorithms are known to be sensitive to hardware impairments [20]. Beyond these factors, the number of antenna elements imposes a fundamental limit on the maximum number of resolvable paths, which may significantly degrade performance in dense multipath environments. Rather than attempting to bypass these physical limits through resolution, the strategy adopted in this paper treats estimation uncertainty as a valuable input for the subsequent localization stage. A comparative analysis of this system-level philosophy against classical algorithm-centric baselines is summarized in Table 1.

#### 2.1.3. Quality-Aware Localization

Building on this estimation strategy, the final stage of the framework involves translating angular measurements into spatial coordinates. In real-world indoor deployments, the presence of non-line-of-sight (NLoS) signals and multipath-induced outliers necessitates a quality-aware approach rather than a simple geometric intersection.

Historically, prior research has addressed measurement reliability primarily through using external signal metrics and statistical filtering. Many frameworks rely on RSS or SNR as proxies for measurement quality, using them to weight the contribution of each link in a WLS or ML estimator. However, in indoor environments, SNR is often decoupled from angular accuracy, as a strong reflection can produce a high-SNR signal with a significantly biased AoA. Modern approaches have moved toward soft information-based localization, which replaces single-value estimates with probabilistic representations to better capture environmental uncertainty [21]. Other specialized techniques, such as the WiCo framework [22], utilize spectrum confidence estimation to identify and down-weight outliers iteratively. While these methods significantly improve accuracy in harsh propagation conditions, they often increase the computational burden or require complex neural network training that may be impractical for low-power deployments.

The framework proposed in this paper advances the state-of-the-art by utilizing a tightly coupled confidence metric derived from the phase-fitting residuals of the principal eigenvector. Unlike external metrics or iterative post-processing, the proposed metric provides a direct, low-complexity assessment of the wavefront planarity. By embedding this “soft information” directly into the WLS cost function, the localization process becomes inherently resilient to multipath distortion without requiring the high-resolution path decomposition of subspace methods. This positions the proposed method as a theoretically principled yet lightweight solution specifically optimized for the hardware and latency constraints of 5G RedCap devices.

### 2.2. Positioning in 2D Space

#### 2.2.1. 1D AoA Estimation

**Figure 1:**
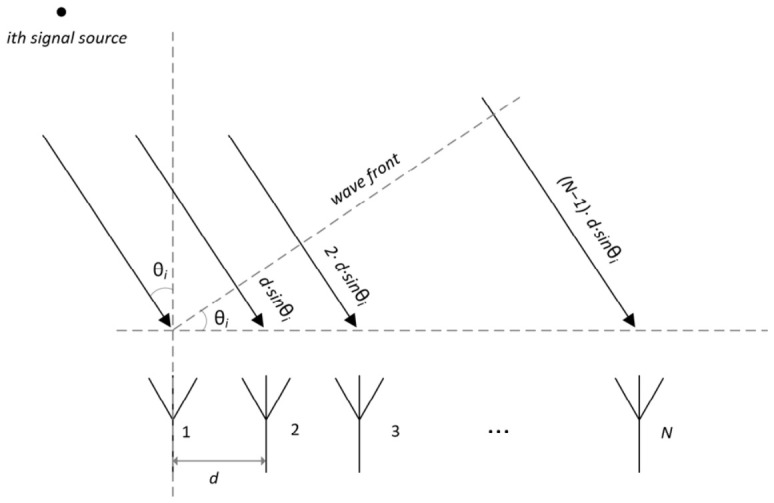
2D signal model and geometric configuration of the antenna array.

Assume a TRP is equipped with an ULA consisting of *N* identical isotropic elements with uniform inter-element spacing *d* (Figure 1). Let *J* be the number of narrowband plane waves, centered at a carrier frequency *f*_c_ (with a corresponding wavelength λ*_c_*), impinging on the array from directions $\left\{\theta_{1},\theta_{2},\ldots ,\theta_{J}\right\}$. The *i*th wave is associated with the *i*th of *J* signal sources whose AoA is denoted as $\theta_{i}$. As illustrated in Figure 1, the AoA is defined as the angle between the broadside of the array (the line orthogonal to the array axis) and the direction of the impinging wave’s propagation.

The response of the antenna array to a plane wave impinging at an angle θ is defined by the steering vector $F\left(\theta \right)$ [23]. Accordingly, we define the *N* × *J* steering matrix, Φ, whose columns are the steering vectors for angles $\left\{\theta_{1},\theta_{2},\ldots ,\theta_{J}\right\}$:

(1) $$\Phi =\left[F\left(\theta_{1}\right)\;F\left(\theta_{2}\right)\ldots F\left(\theta_{J}\right)\right]$$

It is apparent that the steering matrix Φ characterizes the information regarding the angular positions of the signal sources. Consequently, by identifying the components of Φ, one can derive the estimates of the AoAs.

In the case of a ULA, the steering vector takes the following simple form:

(2) $$F\left(\theta_{i}\right)={\left[1e^{-jk_{i}}\;e^{-j2k_{i}}\ldots e^{-j(N-1)k_{i}}\right]}^{T}$$

where the superscript ‘*T*’ indicates the transpose. The parameter $k_{i}$ is defined as:

(3) $$k_{i}=\frac{2\pi d}{\lambda_{c}}\sin\theta_{i}=2\pi d_{\lambda }\sin\theta_{i}$$

where $d_{\lambda }$ represents the normalized inter-element spacing in units of wavelength.

Assuming the *i*th source corresponds to the received signal $s_{i}\left(t\right)$, the array output vector can be expressed as:

(4) $$X\left(t\right)=\Phi S\left(t\right)+N(t)$$

where $S\left(t\right)={\left[s_{1}\left(t\right)\;s_{2}\left(t\right)\ldots s_{J}\left(t\right)\right]}^{T}$ is the signal vector and $N\left(t\right)={\left[n_{1}\left(t\right)\;n_{2}\left(t\right)\ldots n_{N}\left(t\right)\right]}^{T}$ is the additive noise vector.

Based on this model, the spatial covariance matrix of the received signal vector can be defined as:

(5) $$M=E\left\{X\left(t\right){X\left(t\right)}^{H}\right\}$$

where $E\left\{\cdot \right\}$ denotes the expectation operator and the superscript ‘*H*’ indicates the Hermitian transpose.

Under the assumption that the noise is spatially white and uncorrelated with the signals, it follows from (5) that

(6) $$M=\Phi P\Phi^{H}+\sigma^{2}I$$

where $\sigma^{2}$ is the noise variance, **I** is the *N* × *N* identity matrix. The *J* × *J* matrix **P** represents the source covariance matrix, defined as:

(7) $$P=E\left\{S\left(t\right){S\left(t\right)}^{H}\right\}$$

The *i*th diagonal element of matrix **P** represents the power of the signal $s_{i}\left(t\right)$. In the event that the received signals are pairwise uncorrelated, **P** becomes a diagonal matrix.

Being a Hermitian matrix, the spatial covariance matrix M can be represented by its spectral decomposition:

(8) $$M=\sum_{i=1}^{N}\lambda_{i}U_{i}U_{i}^{H}$$

where $\lambda_{i}$ is the *i*th eigenvalue and $U_{i}$ is the corresponding *i*th eigenvector of the matrix **M**.

If the number of antenna elements exceeds the number of signals (i.e., *N* > *J*) and the steering matrix is of full rank, the spectral decomposition in (8) can be partitioned into two distinct components. The first part consists of eigenvectors corresponding to eigenvalues equal to the noise variance, thereby spanning the noise subspace. The second part corresponds to the remaining *J* larger eigenvalues, which span the signal subspace:

(9) $$M=E_{s}\Lambda_{s}E_{s}^{H}+\sigma^{2}E_{n}E_{n}^{H}$$

where $E_{s}=\left[U_{1},\;U_{2},\ldots U_{J}\right]$ and $E_{n}=\left[U_{J+1},\;U_{J+2},\ldots U_{N}\right]$ are the matrices, whose columns consist of the signal and noise eigenvectors, respectively. The *J* × *J* matrix $\Lambda_{s}=diag\left[\lambda_{1},\;\lambda_{2},\ldots \lambda_{J}\right]$ is the diagonal matrix containing the corresponding signal eigenvalues.

By exploiting the orthonormality of the eigenvectors, Equation (9) can be transformed into:

(10) $$M=E_{s}\left(\Lambda_{s}-\sigma^{2}\right)E_{s}^{H}+\sigma^{2}I$$

In the case where only a single source exists (i.e., *J* = 1), Equation (6) reduces to:

(11) $$M=P_{1}F\left(\theta_{1}\right){F\left(\theta_{1}\right)}^{H}+\sigma^{2}I$$

where $P_{1}$ represents the power of the signal $s_{1}\left(t\right)$. Furthermore, Equation (10) can be simplified as follows:

(12) $$M=\left(\lambda_{1}-\sigma^{2}\right)U_{1}U_{1}^{H}+\sigma^{2}I$$

where $\lambda_{1}$ is the signal eigenvalue and $U_{1}$ is the corresponding eigenvector.

By comparing (11) and (12), it can be concluded that in the single-source case, the steering vector $F\left(\theta_{1}\right)$ and the principal eigenvector $U_{1}$ of the spatial covariance matrix M coincide, differing only by a scaling factor. Since eigenvectors are defined with a unit *L*^2^-norm and considering the structure of the steering vector defined in (2) and (3), the relationship can be expressed as:

(13) $$U_{1}=\frac{1}{\sqrt{N}}F\left(\theta_{1}\right)=\frac{1}{\sqrt{N}}\left[\begin{array}{c} 1\\ e^{-j2\pi d_{\lambda }\sin\theta_{1}}\\ \ldots \\ e^{-j2\pi (i-1)d_{\lambda }\sin\theta_{1}}\\ \ldots \\ e^{-j2\pi (N-1)d_{\lambda }\sin\theta_{1}} \end{array}\right]$$

It is straightforward to see from (13) that the phase difference Δ between any two adjacent elements of the eigenvector $U_{1}$ is given by:

(14) $$\Delta =∠\left({U_{1}}_{i}\right)-∠\left({U_{1}}_{i-1}\right)=\varphi_{i}-\varphi_{i-1}=-2\pi d_{\lambda }\sin\theta_{1}$$

where ${U_{1}}_{i}$ denotes the *i*th element of $U_{1}$ and $\varphi_{i}$ is its corresponding phase.

**Figure 2:**
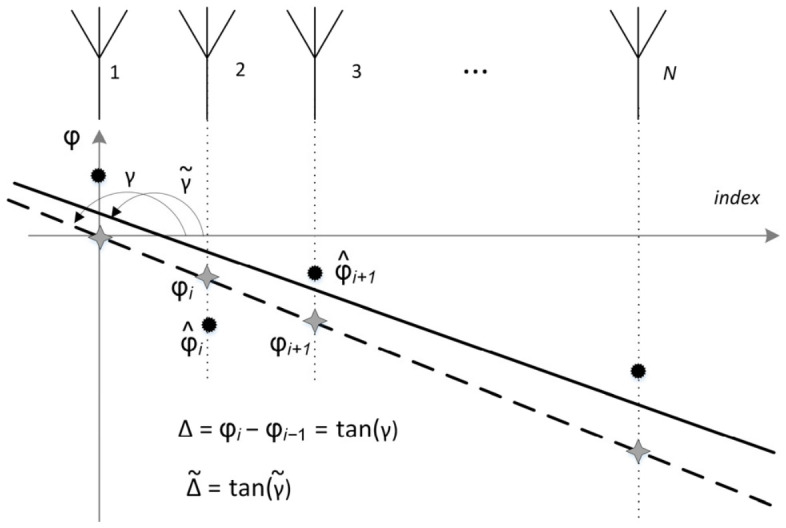
Phase progression of the principal eigenvector elements for the ideal Line-of-Sight (LoS) case (*J* = 1, gray stars for exact values, dashed line for linear approximation) and the multipath scenario (*J* > 1, black circles for exact values, solid line for linear approximation).

Thus, the locus of $∠\left({U_{1}}_{i}\right)$ is a straight line with a slope equal to Δ (represented by the dashed line in Figure 2). Based on this relationship, the estimate for the AoA ${\hat{\theta }}_{1}$ is directly obtained as:

(15) $${\hat{\theta }}_{1}=-\sin^{-1}\left(\frac{\Delta }{2\pi d_{\lambda }}\right)$$

In practice, the phase difference Δ is calculated using a standard phase-unwrapping procedure to resolve potential 2π ambiguities. Furthermore, as follows from (15), the obtained AoA estimate is inherently ambiguous. The maximum angular support provided by a ULA with isotropic elements is limited to a half-plane, i.e., ${\hat{\theta }}_{1}\in \left[-90{}^{\circ},90{}^{\circ}\right]$ provided that $d_{\lambda }\leq 0.5$. In practice, however, this ambiguity can be readily resolved, for example, by utilizing non-isotropic elements with specific radiation patterns. In the context of 5G networks, this is often inherently addressed by the sectorized deployment of TRPs, where each array is designed to cover a specific angular sector (e.g., 120°), thereby naturally restricting the AoA to the unambiguous region.

A more fundamental challenge is that (15) is valid only for the single-source case. In a communications context, this implies ideal Line-of-Sight (LoS) conditions, which assume the absence of multipath propagation. Such conditions are improbable for the majority of 5G deployments, particularly in InF scenarios. Conversely, under more realistic LoS-plus-multipath conditions, one may expect a considerable number of indirect paths, each acting as a distinct signal source. Each of these sources contributes a corresponding signal eigenvalue and signal eigenvector to the spatial covariance matrix.

Assuming the direct path yields the strongest signal, it corresponds to the largest eigenvalue. The related signal eigenvector is defined as the principal eigenvector $U_{1}$. It can be shown that, provided the sources are uncorrelated, any signal eigenvector is a linear combination of the steering vectors. Consequently, the locus of $∠\left({U_{1}}_{i}\right)$ deviates from a straight line, and equality (14) no longer holds.

Nevertheless, one can identify the principal eigenvector $U_{1}$ of the spatial covariance matrix M and approximate its phase progression with a straight line (represented by the solid line in Figure 2). This is achieved by formulating the task as a linear regression problem. In other words, a functional dependent on two parameters (representing the slope and the intercept) must be minimized:

(16) $$f\left(\tilde{\Delta },\;\beta \right)=min\left\{\sum_{i=1}^{N}{\left({\hat{\varphi }}_{i}-{\tilde{\varphi }}_{i}\right)}^{2}\right\}=min\left\{\sum_{i=1}^{N}\varepsilon_{i}^{2}\right\}=min\left\{{\left\|E\right\|}^{2}\right\}$$

where ${\hat{\varphi }}_{i}$ represents the unwrapped phase of the *i*th element of principal eigenvector $U_{1}$ and ${\tilde{\varphi }}_{i}$ is the corresponding phase value derived from the linear approximation. The operator $\left\|\cdot \right\|$ denotes the *L*^2^-norm and **E** is the residual error vector composed of the individual approximation errors $\varepsilon_{i}$.

The functional in (16) reaches its minimum for the parameter vector:

(17) $$\left[\begin{array}{c} \tilde{\Delta }\\ \beta \end{array}\right]={\left(C^{T}C\right)}^{-1}C^{T}\left[\begin{array}{c} {\hat{\varphi }}_{1}\\ {\hat{\varphi }}_{2}\\ \ldots \\ {\hat{\varphi }}_{N} \end{array}\right]$$

where the observation matrix **C** is defined as:

(18) $$C=\left[\begin{array}{cc} 1 & 1\\ 2 & 1\\ \ldots & \ldots \\ N & 1 \end{array}\right]$$

By replacing the slope Δ in (15) with its approximation $\tilde{\Delta }$ derived from (17), the required estimate for the LoS AoA is obtained as:

(19) $${\hat{\theta }}_{LoS}=-\sin^{-1}\left(\frac{\tilde{\Delta }}{2\pi d_{\lambda }}\right)$$

Equation (19) provides a straightforward method for 1D AoA estimation in the presence of multipath. Similar to the Bartlett beamformer, this estimator lacks super-resolution capability. However, it naturally yields a quality indicator for the AoA estimate, or more concisely, a link quality metric.

It is straightforward to see that the AoA estimate provided by the proposed algorithm coincides with the true AoA under ideal LoS conditions. In such a case, the residual approximation errors $\varepsilon_{i}$ are zero, and the functional in (16) reaches its absolute minimum of zero. Consequently, the *L*^2^-norm of the error vector **E** serves as an indicator of how closely the UE-to-TRP communication link approximates the favorable propagation conditions required for high-quality AoA estimation.

It follows from (16) and (17) that the residual error vector **E** can be expressed as:

(20) $$E=\left[\begin{array}{c} {\hat{\varphi }}_{1}\\ {\hat{\varphi }}_{2}\\ \ldots \\ {\hat{\varphi }}_{N} \end{array}\right]-C\left[\begin{array}{c} \tilde{\Delta }\\ \beta \end{array}\right]$$

Considering that the number of antenna elements affects the *L*^2^-norm of vector **E** and to account for the variability in antenna array configurations, it is appropriate to define the link quality metric as the normalized norm:

(21) $$\chi =\sqrt{\frac{1}{N}\sum_{i=1}^{N}\varepsilon_{i}^{2}}=\frac{\left\|E\right\|}{\sqrt{N}}$$

where χ represents the Root Mean Square (RMS) phase error across the array. As defined by (21), a higher value of the link quality metric represents a greater penalty, effectively signaling low confidence in the corresponding AoA estimate.

Crucially, in scenarios where the LoS path is blocked, the principal eigenvector may align with a dominant reflection, potentially leading to a significant AoA estimation error. However, such blocked-LoS cases are typically characterized by strong multipath interference. Consequently, similar to severe multipath LoS scenarios, the resulting NLoS AoA estimate will be associated with a high-value link quality metric, signaling a reduction in reliability. This inherently down-weights the contribution of that TRP within the WLS estimator, effectively preserving the overall localization accuracy by prioritizing TRPs with clearer propagation paths.

It should be emphasized that (5) defines the true spatial covariance matrix, assuming it is perfectly known. In practice, however, the true matrix is unavailable, and one must operate with an estimate. Numerous approaches exist for estimating the spatial covariance matrix, including adaptations for correlated sources [19,24,25,26]. These methods typically derive the estimate from a finite number of time-domain snapshots. A straightforward yet effective approach is to utilize the sample covariance matrix, $\hat{M}$, which represents the unstructured maximum likelihood estimate of the spatial covariance matrix.

Since 5G NR utilizes OFDM signals, frequency-domain channel estimation is typically more practical than time-domain methods [27]. In practice, the spatial covariance matrix is estimated using a finite number of channel estimates obtained from a set of pilot subcarriers. Analogous to the time-domain approach, the frequency-domain sample covariance matrix can be employed as the spatial covariance estimate:

(22) $$\hat{M}={diag\left(\tilde{M}\right)}^{-1/2}\tilde{M}\;{diag\left(\tilde{M}\right)}^{-1/2}$$

where $\tilde{M}$ is the unnormalized sample covariance matrix defined as:

(23) $$\tilde{M}=\frac{1}{L}\sum_{l=1}^{L}\hat{h}\left(l\right)\hat{h}{\left(l\right)}^{H}$$

In these expressions, *L* denotes the number of pilot subcarriers used for channel estimation and $\hat{h}\left(l\right)$ is the *N* × 1 channel estimate vector across *N* receiving antennas.

Consequently, to perform AoA estimation, specifically to apply the estimator (19) and derive the link quality metric (21), the principal eigenvector of the true spatial covariance matrix is replaced by the corresponding eigenvector of the normalized sample covariance matrix $\hat{M}$ defined in (22).

#### 2.2.2. UE Localization in 2D Space

Assume that positioning is performed using *K* TRPs with known coordinates. In the 2D space under consideration, the position of the *p*th TRP is defined by the coordinates $\left(x_{p},\;y_{p}\right)$. These *K* UE-to-TRP links provide a set of AoA estimates, {${\hat{\theta }}_{p}$}, which are defined within a global coordinate system. The objective is to estimate the UE coordinates in 2D space, denoted as $\left({\hat{x}}_{o},\;{\hat{y}}_{o}\right)$.

It is important to note that the AoA estimate (19) is defined within the local coordinate system of the specific antenna array. Therefore, to transform this estimate into the global coordinate system, the orientation of each antenna array is assumed to be precisely known.

**Figure 3:**
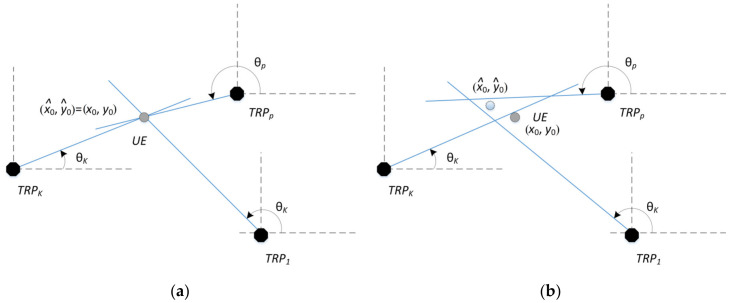
2D AoA-based positioning geometry: (**a**) ideal scenario with error-free angular estimates, where the blue lines of bearing from the three TRPs intersect at a single point (true UE position); (**b**) practical scenario in the presence of estimation errors, where the lines of bearing create an uncertainty region.

It is straightforward to see that each 1D AoA estimate defines a straight line passing through the coordinates of both the TRP and the UE. Under the assumption of perfect AoA estimation, the unique intersection of these *K* lines determines the UE position, $\left({\hat{x}}_{o},\;{\hat{y}}_{o}\right)$, as illustrated in Figure 3a.

Using the point-slope form of a linear equation, the relationship for the *p*th UE-to-TRP link can be expressed as:

(24) $$y-y_{p}=a_{p}\left(x-x_{p}\right)$$

where $a_{p}=\tan{\hat{\theta }}_{p}$ represents the slope of the line determined by the estimated AoA.

Expression (24) facilitates the construction of a system of *K* linear equations:

(25) $$\left\{\begin{array}{c} y=a_{1}x+\left(y_{1}-a_{1}x_{1}\right)\\ \ldots \\ y=a_{p}x+\left(y_{p}-a_{p}x_{p}\right)\\ \ldots \\ y=a_{K}x+\left(y_{K}-a_{K}x_{K}\right) \end{array}\right.$$

Alternatively, this system can be expressed in the following matrix form:

(26) $$\left[\begin{array}{cc} -a_{1} & 1\\ -a_{2} & 1\\ \ldots & \ldots \\ -a_{K} & 1 \end{array}\right]\left[\begin{array}{c} x_{0}\\ y_{0} \end{array}\right]=\left[\begin{array}{c} y_{1}-a_{1}x_{1}\\ y_{2}-a_{2}x_{2}\\ \ldots \\ y_{K}-a_{K}x_{K} \end{array}\right]⟺A\left[\begin{array}{c} x_{0}\\ y_{0} \end{array}\right]=b$$

If only two TRPs are available (*K* = 2), the system in (25) consists of two equations and yields a single unique solution, provided the equations are consistent. This implies that, theoretically, AoA-based positioning can be performed using only two TRPs. For *K* > 2, the system in (25) becomes an overdetermined system of linear equations. In general, such a system has no exact solution due to measurement noise and multipath.

However, assuming that matrix **A** is of full rank, an approximate solution can be derived using the Moore–Penrose pseudoinverse, which corresponds to the Ordinary Least Squares (OLS) estimator [28]:

(27) $$\left[\begin{array}{c} {\hat{x}}_{o}\\ {\hat{y}}_{o} \end{array}\right]={\left(A^{T}A\right)}^{-1}A^{T}b$$

In the case where all AoAs are estimated perfectly (i.e., the error-free scenario), the solution provided by (27) coincides with the true UE position, as illustrated in Figure 3a. In practice, however, the accuracy of AoA estimation is finite. Consequently, the position estimate deviates from the true UE coordinates, resulting in a positioning error (see Figure 3b). Generally, this positioning error depends on both the AoA estimation accuracy and the TRP deployment geometry, the latter of which is characterized by the Geometric Dilution of Precision (GDOP).

The primary drawback of the OLS estimator (27) is that it does not incorporate information regarding the quality of the AoA estimates. To address this, one can apply additional conditioning to the available estimates based on the link quality metric (21). While a rigid link selection achieved by selecting a subset of the “best” links (those with the smallest metric values) out of the *K* available links is possible, a soft link selection approach is generally superior. This is realized by incorporating a weighting matrix into the LS estimator, which provides a more robust solution.

Following this strategy, the UE position estimate can be determined using the Weighted Least Squares (WLS) estimator:

(28) $$\left[\begin{array}{c} {\hat{x}}_{o}\\ {\hat{y}}_{o} \end{array}\right]={\left(A^{T}WA\right)}^{-1}A^{T}Wb$$

where the *K* × *K* weighting matrix **W** is a diagonal matrix defined as:

(29) $$W=\frac{1}{\sum_{p=1}^{K}\chi_{p}^{-2}}\left[\begin{array}{cccc} \chi_{1}^{-2} & 0 & \ldots & 0\\ 0 & \chi_{2}^{-2} & \ldots & 0\\ \ldots & \ldots & \ldots & 0\\ 0 & 0 & 0 & \chi_{K}^{-2} \end{array}\right]$$

In this expression, $\chi_{p}$ represents the link quality metric of the *p*th UE-to-TRP link, calculated according to (21). While the scalar normalization of the matrix does not alter the final estimate, it keeps the weights within a [0, 1] relative range.

According to the Gauss–Markov theorem, the optimal weights for a WLS estimator are the inverse of the measurement variances. In the proposed framework, the link quality metric (21) represents the RMS phase error across the array, which serves as a physical proxy for the standard deviation of the AoA estimate. Consequently, since the variance of each estimate is proportional to $\chi_{p}^{2}$, the choice of weights in (29) is statistically justified.

This grounded weighting scheme ensures that the localization algorithm dynamically prioritizes high-confidence, quasi-LoS links while heavily penalizing measurements affected by significant phase distortions. As demonstrated in Section 3, this approach provides the system-level robustness required to maintain reliable positioning in high-multipath or NLoS environments where individual AoA estimates may be compromised.

### 2.3. Positioning in 3D Space

#### 2.3.1. 2D AoA Estimation

**Figure 4:**
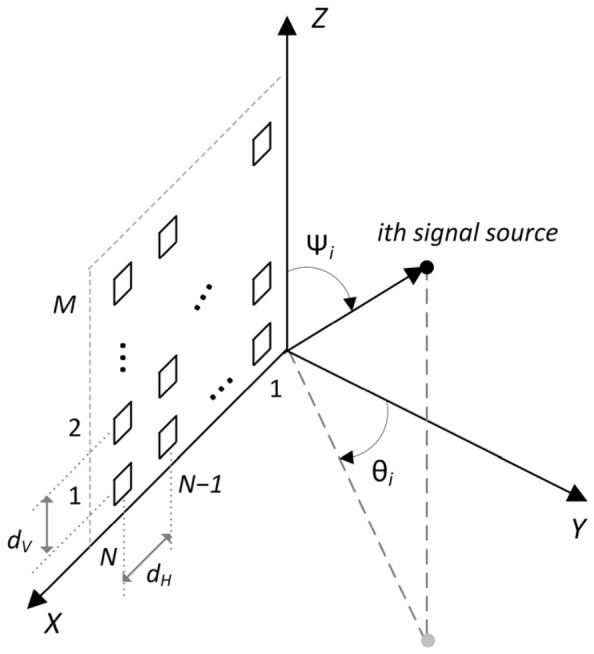
3D signal model and geometric configuration of the uniform rectangular array (URA).

The 2D positioning problem discussed previously relies on 1D AoA estimation. In contrast, positioning in 3D physical space necessitates 2D AoA estimation, requiring the simultaneous estimation of both the azimuth angle $\theta_{i}$ and the zenith angle $\psi_{i}$ (see Figure 4). It is important to highlight that the 2D AoA estimation problem can be decoupled into two independent 1D AoA estimation tasks. Specifically, the azimuth and zenith angles can be estimated separately by exploiting the orthogonal geometry of the antenna array.

While various antenna configurations exist, it is appropriate to generalize the ULA concept by considering a Uniform Rectangular Array (URA). In this case, the 1D AoA estimation results presented in Section 2.2.1 remain applicable, requiring only minor modifications to accommodate the two-dimensional spatial sampling.

Assume a TRP is equipped with a URA consisting of *N* identical isotropic elements with an inter-element spacing $d_{H}$ in the horizontal plane, and *M* identical isotropic elements with an inter-element spacing $d_{V}$ in the vertical plane (see Figure 4). It is evident that the URA comprises *M* × *N* elements, organized into *M* rows and *N* columns.

Each row and column of the URA can be treated as a separate horizontal or vertical ULA. Consequently, the horizontal spatial covariance matrix can be estimated by averaging the sample covariance matrices across all *M* rows. Using Equation (23), this is expressed as:

(30) $${\tilde{M}}_{H}=\frac{1}{M}\sum_{i=1}^{M}{\tilde{M}}_{i}=\frac{1}{M}\sum_{i=1}^{M}\left\{\frac{1}{L}\sum_{l=1}^{L}{\hat{h}}_{i}\left(l\right){{\hat{h}}_{i}\left(l\right)}^{H}\right\}$$

where *L* denotes the number of subcarriers utilized for channel estimation and ${\hat{h}}_{i}\left(l\right)$ is the *N* × 1 channel estimate vector for the *i*th antenna row.

Similarly, the spatial covariance matrix for the vertical plane can be estimated by averaging the sample covariance matrices across all *N* columns:

(31) $${\tilde{M}}_{V}=\frac{1}{N}\sum_{j=1}^{N}{\tilde{M}}_{j}=\frac{1}{N}\sum_{j=1}^{N}\left\{\frac{1}{L}\sum_{l=1}^{L}{\hat{h}}_{j}\left(l\right){{\hat{h}}_{j}\left(l\right)}^{H}\right\}$$

where ${\hat{h}}_{j}\left(l\right)$ is the *M* × 1 vector channel estimate vector for the *j*th antenna column.

To perform the 2D AoA estimation, specifically to derive the vertical and horizontal phase slopes ${\tilde{\Delta }}_{H}$ and ${\tilde{\Delta }}_{V}$, the principal eigenvectors are extracted from the normalized versions of the spatial covariance matrices ${\hat{M}}_{H}$ and ${\hat{M}}_{V}$, following the procedure described in (22). By applying the linear regression framework of Section 2.2.1 to these eigenvectors, the estimates for the zenith angle ${\hat{\psi }}_{LoS}$ and the azimuth angle ${\hat{\theta }}_{LoS}$ relative to the coordinate system in Figure 4 are obtained as:

(32) $${\hat{\psi }}_{LoS}=\frac{\pi }{2}-\sin^{-1}\left(\frac{{\tilde{\Delta }}_{V}}{2\pi d_{\lambda V}}\right)$$

(33) $${\hat{\theta }}_{LoS}=\sin^{-1}\left(\frac{{\tilde{\Delta }}_{H}}{2\pi d_{\lambda H}\sin{\hat{\psi }}_{LoS}}\right)$$

The parameters $d_{\lambda V}$ and $d_{\lambda H}$ represent the inter-element spacings $d_{V}$ and $d_{H}$ in units of wavelength. The inclusion of the $\sin{\hat{\psi }}_{LoS}$ term in (33) accounts for the geometric scaling of the effective horizontal aperture as a function of the signal’s vertical inclination.

In addition to the 2D AoA estimates, the link quality metrics can be readily determined for both the horizontal and vertical planes:

(34) $$\chi_{H}=\frac{\left\|E_{H}\right\|}{\sqrt{N}}$$

(35) $$\chi_{V}=\frac{\left\|E_{V}\right\|}{\sqrt{M}}$$

where $E_{H}$ and $E_{V}$ are the residual error vectors derived from the linear phase approximations in the horizontal and vertical dimensions, respectively.

In practice, it is beneficial to utilize a single metric to reflect the overall link quality. Such a metric can be obtained by combining (34) and (35). By simply aggregating the individual components, the total link quality metric $\chi_{\Sigma }$ is defined as:

(36) $$\chi_{\Sigma }=\chi_{H}+\chi_{V}$$

This additive aggregation provides a holistic assessment of the wavefront consistency across the entire 2D aperture, ensuring that a TRP link is penalized if significant phase distortion is detected in either the horizontal or vertical dimension. Furthermore, the additive aggregation in (36) provides superior outlier rejection compared to a standard root sum of squares combination. By increasing the effective penalty gradient within the WLS weight calculation, the additive metric more aggressively de-prioritizes TRP links affected by significant phase distortion in either the horizontal or vertical aperture.

Consequently, the implementation of 2D AoA estimation involves constructing the spatial covariance matrices (30) and (31), normalizing them, extracting their principal eigenvectors and subsequently applying the estimators defined in (32) and (33). The link quality metric (36) is obtained as an intrinsic part of this estimation procedure.

#### 2.3.2. UE Localization in 3D Space

Assume that positioning is performed using *K* TRPs with known coordinates. In the 3D space under consideration, the position of the *p*th TRP is defined by the coordinates $\left(x_{p},\;y_{p},z_{p}\right)$. These *K* UE-to-TRP links provide a set of 2D AoA estimates, {${\hat{\theta }}_{p},\;{\hat{\psi }}_{p}$}, which are defined within a global coordinate system. The objective is to estimate the UE coordinates in 3D space, denoted as $\left({\hat{x}}_{o},\;{\hat{y}}_{o},\;{\hat{z}}_{o}\right)$.

The UE coordinates $\left({\hat{x}}_{o},\;{\hat{y}}_{o},\;{\hat{z}}_{o}\right)$ can be estimated using several approaches. One method involves decoupling the 3D problem into two 2D tasks; for example, by considering the XY and XZ planes separately to solve for the coordinate pairs $\left({\hat{x}}_{o},\;{\hat{y}}_{o}\right)$ and $\left({\hat{x}}_{o},\;{\hat{z}}_{o}\right)$. Alternatively, a successive estimation approach can be employed where the horizontal coordinates $\left({\hat{x}}_{o},\;{\hat{y}}_{o}\right)$ are determined in the first step. Given the known TRP positions and the available zenith angle estimates, the vertical coordinate ${\hat{z}}_{o}$ can then be directly recovered for each UE-to-TRP link.

Nevertheless, the 3D positioning problem can be addressed using a unified approach based on the generalized parametric representation of a straight line in $R^{3}$. By leveraging projective geometry and the WLS framework, the 3D UE position can be estimated as [29]:

(37) $$\left[\begin{array}{c} {\hat{x}}_{o}\\ {\hat{y}}_{o}\\ {\hat{z}}_{o} \end{array}\right]={\left(\sum_{p=1}^{K}W_{p}U_{p}^{⊥}\right)}^{-1}\sum_{p=1}^{K}W_{p}U_{p}^{⊥}\left[\begin{array}{c} x_{p}\\ y_{p}\\ z_{p} \end{array}\right]$$

where $W_{p}$ is the 3 × 3 weighting matrix for the *p*th UE-to-TRP link and $U_{p}^{⊥}=I-u_{p}u_{p}^{T}$ is the 3 × 3 rank-2 symmetric idempotent projection matrix. This matrix projects any vector onto the plane orthogonal to the unit direction vector $u_{p}$. The vector $u_{p}$ is determined by the 2D AoA estimate {${\hat{\theta }}_{p},\;{\hat{\psi }}_{p}$} as follows:

(38) $$u_{p}=\left[\begin{array}{c} \cos{\hat{\theta }}_{p}\sin{\hat{\psi }}_{p}\\ \sin{\hat{\theta }}_{p}\sin{\hat{\psi }}_{p}\\ \cos{\hat{\psi }}_{p} \end{array}\right]$$

The solution (37) can be efficiently implemented using a block-matrix representation:

(39) $$\left[\begin{array}{c} {\hat{x}}_{o}\\ {\hat{y}}_{o}\\ {\hat{z}}_{o} \end{array}\right]={\left({U^{⊥}}^{T}WU^{⊥}\right)}^{-1}{U^{⊥}}^{T}W\rho$$

where matrix $U^{⊥}$ is a 3*K* × 3 matrix composed of the individual projection matrices $U_{p}^{⊥}$:

(40) $$U^{⊥}=\left[\begin{array}{c} U_{1}^{⊥}\\ \ldots \\ U_{K}^{⊥} \end{array}\right]$$

The 3*K* × 3*K* block diagonal matrix W consists of *K* symmetric positive-definite weighting matrices:

(41) $$W=\left[\begin{array}{cccc} W_{1} & 0 & \ldots & 0\\ 0 & W_{2} & \ldots & \ldots \\ \ldots & \ldots & \ldots & \ldots \\ 0 & \ldots & \ldots & W_{K} \end{array}\right]$$

and the 3*K* × 1 vector ρ is formed by the concatenated coordinates of the TRPs:

(42) $$\rho =\left[\begin{array}{c} \left[\begin{array}{c} x_{1}\\ y_{1}\\ z_{1} \end{array}\right]\\ \ldots \\ \left[\begin{array}{c} x_{K}\\ y_{K}\\ z_{K} \end{array}\right] \end{array}\right]$$

It is evident that while the WLS estimator (39) requires a 3 × 3 weighting matrix for each UE-to-TRP link, the link quality metric derived in (36) is a scalar value. To satisfy this requirement, the weighting matrix for the *p*th link can be defined as:

(43) $$W_{p}=\frac{1}{\sum_{p=1}^{K}{\chi_{\Sigma }}_{p}^{-2}}\left[\begin{array}{ccc} {\chi_{\Sigma }}_{p}^{-2} & 0 & 0\\ 0 & {\chi_{\Sigma }}_{p}^{-2} & 0\\ 0 & 0 & {\chi_{\Sigma }}_{p}^{-2} \end{array}\right]$$

#### 2.3.3. Practical Deployment Considerations

Crucially, the proposed uplink-based framework offers inherent advantages regarding system stability. While global positioning accuracy depends on the accurate orientation of the TRPs within the network coordinate system, these fixed infrastructure points are typically assumed to be precisely aligned during the network installation phase. The assumption of high TRP density is consistent with 5G 3GPP infrastructure standards for industrial use cases.

To ensure architectural feasibility within standard cellular networks, the localization process partitions computational tasks across distinct entities, aligning explicitly with the 3GPP Location Management Function (LMF) topology:

- The UE side: The resource-constrained RedCap UE transmits standard uplink Sounding Reference Signals (SRS). It performs no positioning computations, successfully preserving its single-antenna configuration and low-power characteristics.
- The TRP side: Each TRP captures the uplink SRS, estimates the frequency-domain sample covariance matrix, extracts the principal eigenvector, and computes its localized 2D AoA estimate {${\hat{\theta }}_{p},\;{\hat{\psi }}_{p}$} alongside its localized link quality metric ${\chi_{\Sigma }}_{p}$.
- The Network side (LMF): Crucially, individual TRPs do not communicate with each other. Instead, they forward the estimated local angles and associated link quality indicators up to a centralized network entity known as the LMF via the 5G Next Generation Application Protocol (NG-AP) control plane. The unified 3D WLS block-matrix inversion derived in (39) is executed entirely at the centralized LMF, which maintains the global coordinates of all fixed TRPs. This completely eliminates any complex routing overhead between TRPs.

The residual systematic errors as well as inevitable hardware-level array calibration errors are treated by the proposed framework not as fatal flaws, but as sources of phase distortion. By quantifying the RMS phase deviation from a theoretical linear model, the proposed metric (36) is designed to identify and de-prioritize TRP links suffering from significant inter-element phase variations or mutual coupling, aiming to prevent hardware impairments from disproportionately biasing the localization result.

The assumption of dominant LoS is not a requirement for system operation but a baseline. As demonstrated in Section 3, even when individual TRP links suffer from significant multipath distortion, the system-level redundancy of a dense 5G infrastructure allows the framework to isolate geometrically consistent links. This “self-healing” behavior exploits spatial diversity to maintain localization integrity in industrial environments where mobile machinery frequently obstructs LoS paths.

It is important to note that AoA estimation accuracy generally depends on the value of the true AoA. Typically, peak estimation performance is achieved when the incident signal aligns with the antenna array boresight. Consequently, as the AoA approaches the array end-fire, the precision of the AoA estimates typically degrades. As a result, in practice, it can be beneficial to perform pre-filtering of the metrics used in (43) based on the corresponding AoA estimates.

The pre-filtering approach is formulated by redefining the link quality metric through a conditional mapping. The metric remains unchanged if the estimated azimuth and zenith angles fall within the angular threshold $T_{r}$ relative to the array boresight; otherwise, it is set to infinity:

(44) $${{\tilde{\chi }}_{\Sigma }}_{p}=\left\{\begin{array}{c} {\chi_{\Sigma }}_{p},\;if\;\max\left(\left|{\hat{\theta }}_{p}\right|,\;\left|90{}^{\circ}-{\hat{\psi }}_{p}\right|\right)\;\leq T_{r}\\ \infty ,\;otherwise \end{array}\right.,$$

where $T_{r}$ is the angular threshold defining the reliable estimation range. Since the WLS weights are inversely proportional to the square of the metric, this mapping effectively assigns a zero weight to any TRP link originating from the unreliable end-fire regions of the antenna array.

The threshold $T_{r}$ can be selected based on the physical limitations of the antenna array’s effective aperture. As the angle relative to the array boresight increases, the effective aperture decreases by a factor of $\cos\left(\cdot \right)$. For instance, at 60°, the aperture is reduced by 50%, which significantly increases sensitivity to noise and typically leads to less accurate estimates. Restricting the measurements to this reliable field of view is intended to ensure that primarily high-confidence AoA estimates are utilized for the subsequent localization phase, preventing low-quality measurements from biasing the WLS estimator.

## 3. Experiment and Result Analysis

The evaluation environment adheres to the 3GPP TR 38.901 benchmark, namely InF-SH scenario [7]. This environment represents typical large industrial halls characterized by sparse clutter. As detailed in Table 2, the simulation employs a stochastic channel model that statistically accounts for the presence of industrial equipment and the reflective properties of factory materials.

The clutter within this layout is associated with storage or commissioning areas with open spaces so that clutter density is 40%. While sparse clutter often leads to a more dominant LoS component, the large hall dimensions (300 × 150 × 25 m) are potentially associated with higher positioning errors due to increased path loss for distant TRPs.

According to the specification, 18 TRPs are arranged in a rectangular grid with 50 m spacing. In the considered setup, all TRPs are deployed at a uniform height of 8 m, while the UEs are distributed throughout the horizontal plane at a constant height of 1.5 m. To address the quasi-stationary scenario, the UEs are assumed to be moving at 3 km/h. This velocity ensures that the channel remains coherent over the duration of the pilot signal transmission, allowing for accurate sample covariance estimation while reflecting the typical movement of industrial assets or personnel.

**Figure 5:**
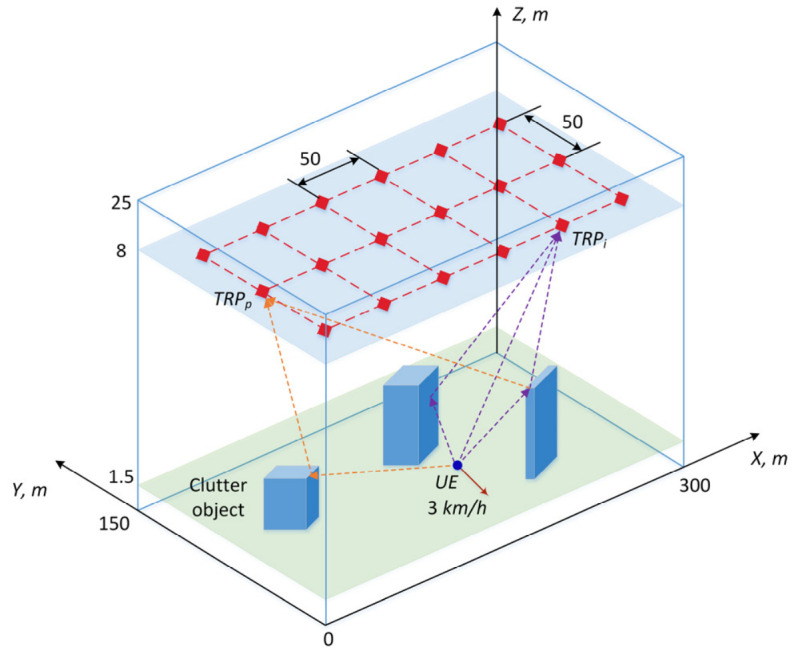
Geometric layout and network topology of the InF-SH simulation environment, including a schematic representation of multipath LoS and NLoS links. The red squares denote the TRP locations, which are interconnected by the red dashed lines to outline the overall network grid deployment.

To provide architectural clarity, the geometric layout and network topology of the simulation environment are illustrated in Figure 5. A conceptual sketch of the multipath LoS and NLoS links is also included.

Crucially, the performance of AoA-based positioning frameworks is highly sensitive to the antenna configuration. The potential TRP antenna array configurations are defined in the 3GPP TR 38.901 specification. To account for varying infrastructure capabilities and to evaluate the trade-off between hardware complexity and the angular resolution required to meet the 3GPP accuracy targets for RedCap UEs, 4 × 4, 8 × 4 and 8 × 8 URAs at the TRP are considered. The inter-element spacings are $d_{\lambda H}=d_{\lambda V}=0.5$. This spacing is specifically chosen to prevent grating lobes, which is vital for the linear phase regression model presented in Section 2.2.1. The UE utilizes a single isotropic antenna and the transmit power is set to 23 dBm.

As previously discussed, 3GPP RedCap UEs are characterized by a system bandwidth up to 20 MHz. To align the simulation setup with 3GPP RedCap UE capabilities, the estimation of the spatial covariance matrix is performed with the SRSs utilizing subcarrier spacing (SCS) either 15 kHz or 30 kHz. Specifically, a Comb-2 structure with two consecutive OFDM symbols is utilized for SRS allocation. The simulation parameters are summarized in Table 2.

It is important to note that for a given system bandwidth, the specific SRS configuration determines the number of pilot subcarriers (parameter *L* in Equations (30) and (31)) and the resulting effective bandwidth for both 15 kHz and 30 kHz SCS. The number of pilot subcarriers *L* presented in Table 3 accounts for the maximum SRS bandwidth configurations permitted by 3GPP standards, which are typically smaller than the total system bandwidth to ensure adequate guard bands. For the considered Comb-2, 2-symbol SRS allocation, this results in a snapshot count *L* that ranges from 96 to 1152, providing a diverse set of conditions to evaluate the framework’s robustness across different RedCap resource allocations.

The performance of the proposed positioning framework is evaluated through the cumulative distribution function of the horizontal and vertical positioning errors. 2D AoA estimation is performed on the uplink using the estimators derived in (32) and (33), while the UE position is determined via the WLS estimator (39), which incorporates the link quality metric defined in (36). The positioning error is defined as the Euclidean distance between the true and estimated UE coordinates.

**Figure 6:**
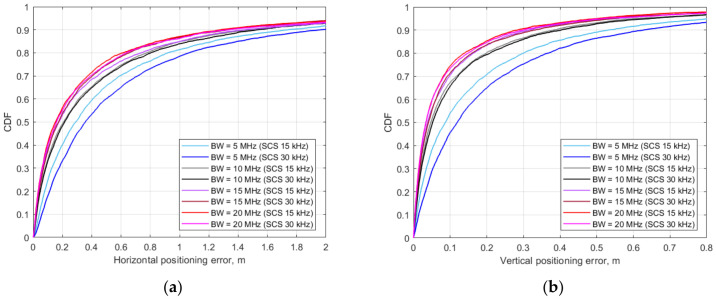
CDF of positioning error for (4, 4) URA configuration: (**a**) horizontal; (**b**) vertical.

**Figure 7:**
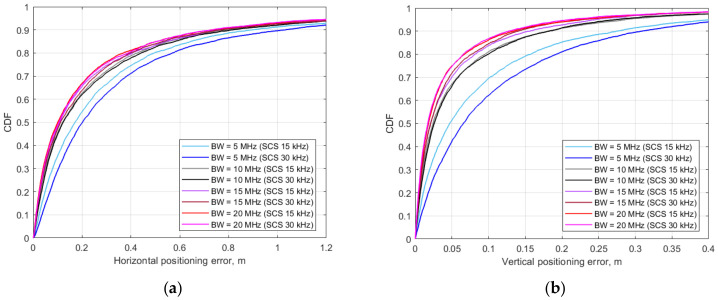
CDF of positioning error for (8, 4) URA configuration: (**a**) horizontal; (**b**) vertical.

**Figure 8:**
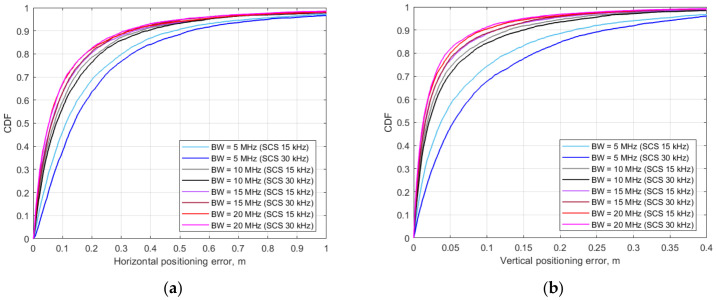
CDF of positioning error for (8, 8) URA configuration: (**a**) horizontal; (**b**) vertical.

Figure 6, Figure 7 and Figure 8 illustrate the positioning accuracy for the (4, 4), (8, 4), and (8, 8) URA configurations, respectively.

The highest precision is achieved with the (8, 8) configuration, providing a baseline for the peak capability. As shown in Figure 8, the 90th percentile horizontal and vertical errors for the 5 MHz/30 kHz ‘worst-case’ scenario are 0.55 m and 0.27 m, respectively. As expected, the 5 MHz/15 kHz case leverages both a larger number of pilot subcarriers and a wider effective bandwidth, enhancing positioning accuracy to 0.48 m and 0.22 m, respectively. These results comfortably satisfy the most stringent 3GPP requirements for industrial RedCap UEs, proving that the proposed AoA-based positioning can provide sub-meter horizontal and vertical accuracy even at the minimum 5 MHz bandwidth.

It is noteworthy that the 10 MHz/30 kHz configuration yields the 90th percentile horizontal and vertical errors of 0.39 m and 0.15 m, respectively. Given that the 10 MHz/30 kHz and 5 MHz/15 kHz cases utilize the same number of pilot subcarriers (*L* = 288), this performance gain is primarily attributable to the increased effective bandwidth. A further improvement is observed in the 10 MHz/15 kHz case, which achieves horizontal and vertical errors of 0.37 m and 0.13 m. This additional gain is driven by the simultaneous increase in both the effective bandwidth and the total number of pilot subcarriers available for spatial covariance estimation.

As seen in Figure 8, further increases in system bandwidth yield only marginal performance enhancements. Specifically, the 15 MHz/15 kHz and 15 MHz/30 kHz configurations, despite utilizing a different number of pilot subcarriers, are characterized by the same effective bandwidth and share 90th percentile horizontal and vertical errors of 0.33 m and 0.11 m, respectively. A similar trend is observed for the 20 MHz ‘best-case’ scenario, where the 90th percentile horizontal and vertical errors reach 0.32 m and 0.09 m. These findings suggest that once a sufficiently effective bandwidth is established for stable channel estimation, the positioning accuracy becomes primarily limited by the angular resolution of the antenna array rather than the frequency-domain resources.

As illustrated in Figure 7, the trends observed for the (8, 8) array remain applicable to the (8, 4) antenna configuration. However, because this configuration features only four elements in the horizontal plane, it is characterized by reduced azimuth resolution. Consequently, the 90th percentile horizontal error for the 5 MHz/30 kHz ‘worst-case’ scenario is 1.03 m, which slightly exceeds the 3GPP industrial requirement. Nevertheless, the 5 MHz/15 kHz configuration, by leveraging a larger number of subcarriers, yields a 90th percentile horizontal error of 0.9 m, successfully satisfying the target. This indicates that while the (8, 4) array is on the edge of industrial compliance at minimum bandwidths, it remains a viable complexity-optimized alternative when slightly more frequency-domain resources are available.

It is noteworthy that while the (8, 4) and (8, 8) configurations share the same zenith aperture, the former utilizes only half the number of antenna columns for spatial averaging. Consequently, the (8, 4) configuration yields slightly reduced vertical accuracy compared to the full (8, 8) array. Specifically, the 5 MHz/30 kHz and 5 MHz/15 kHz cases result in 90th percentile vertical errors of 0.31 m and 0.27 m, respectively. Despite this marginal degradation, the performance remains within the 3.0 m 3GPP vertical requirement.

Similar to the (8, 8) configuration, the positioning performance of the (8, 4) array improves with increased system bandwidth. In the 20 MHz ‘best-case’ scenario, the 90th percentile horizontal and vertical errors reach 0.72 m and 0.13 m, respectively.

The (4, 4) URA represents the baseline for hardware complexity in 3D positioning. As shown in Figure 6, while this configuration does not meet the stringent 1.0 m industrial target, it comfortably satisfies the 3GPP commercial RedCap requirement of 3.0 m. Specifically, the 90th percentile horizontal and vertical errors for the 5 MHz/30 kHz ‘worst-case’ scenario are 1.98 m and 0.64 m, respectively. In the 20 MHz ‘best-case’ scenario, these errors improve to 1.27 m and 0.28 m. This significant performance degradation relative to the (8, 8) array is attributable to the simultaneous reduction in both angular resolution and the spatial averaging capability of the smaller aperture. Nevertheless, these results suggest that a 16-element array remains a cost-effective choice for general asset tracking and commercial use cases where stringent sub-meter precision is not a critical requirement.

Notably, additional evaluations incorporating the angular pre-filtering described in (44) revealed a negative impact on positioning accuracy within the InF-SH deployment. This is attributed to the inherent capability of the WLS estimator to ‘soft-filter’ measurements autonomously. Specifically, as the effective aperture decreases at wide angles, the resulting increase in the link quality metric (36) naturally reduces the corresponding weight in (43). By replacing this adaptive weighting with a hard threshold, the system suffers from an increased GDOP. The exclusion of TRP links (even those with lower angular precision) weakens the geometric stability, outweighing any potential gains from end-fire mitigation.

**Figure 9:**
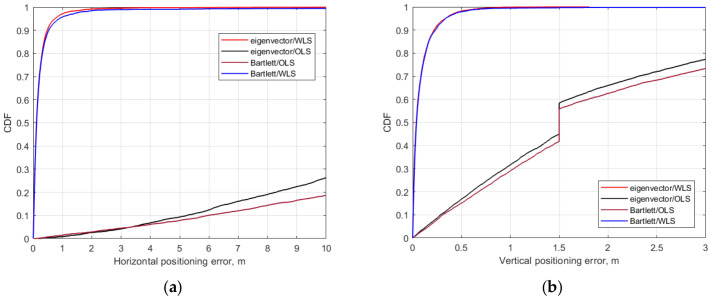
Comparison of eigenvector/WLS with eigenvector/OLS, Bartlett/OLS and Bartlett/WLS frameworks for the (8, 8) URA (5 MHz/15 kHz SCS): (**a**) CDF of horizontal positioning error; (**b**) CDF of vertical positioning error.

To quantify the benefit of the proposed quality-aware weighting and the efficiency of the eigenvector-based AoA estimation, the framework is compared against both an eigenvector/OLS estimator and a Bartlett baseline. The 2D AoA Bartlett estimator is implemented as a sequence of 1D AoA estimators in a manner similar to the eigenvector-based estimator presented in Section 2.3.1. Figure 9 presents this comparison for the (8, 8) URA configuration under the 5 MHz/15 kHz scenario.

As depicted in Figure 9, the proposed WLS framework demonstrates a clear performance advantage over the OLS alternative. While the OLS results are derived from independent realizations of the same AoA estimation process, the estimator treats every UE-to-TRP link with equal weight. Consequently, the OLS approach is highly susceptible to multipath-induced outliers, resulting in a significant positioning error floor. Notably, the vertical error CDF exhibits a distinct plateau at 1.5 m. This behavior stems from the poor vertical observability characteristic of large factory halls. In scenarios where the OLS algorithm fails to resolve the elevation for distant or multipath-affected links, the vertical estimate effectively collapses to the ground plane (*z* = 0). This geometric collapse results in a deterministic vertical error exactly equal to the UE’s height of 1.5 m.

The performance gap between the eigenvector/OLS and Bartlett/OLS curves highlights the superior stability of the eigenvector-based approach. While both estimators perform comparably under high-SNR conditions, the search-based Bartlett method is more susceptible to peak-detection failures in the bandwidth-constrained 5 MHz NLoS regime. In such instances, the Bartlett estimator may produce outlier results (where a fallback mechanism forces the AoA estimate to a nominal boresight value) that significantly skew the final OLS coordinates. In contrast, the principal eigenvector approach utilizes a stable, closed-form phase regression that is inherently immune to search-grid artifacts and peak-merging effects, providing more consistent inputs for localization. This distinction is further validated by the convergence of the eigenvector/WLS and Bartlett/WLS curves. The proposed WLS weighting effectively identifies and suppresses the Bartlett outliers, rendering the final positioning performance less dependent on the underlying estimation method.

**Figure 10:**
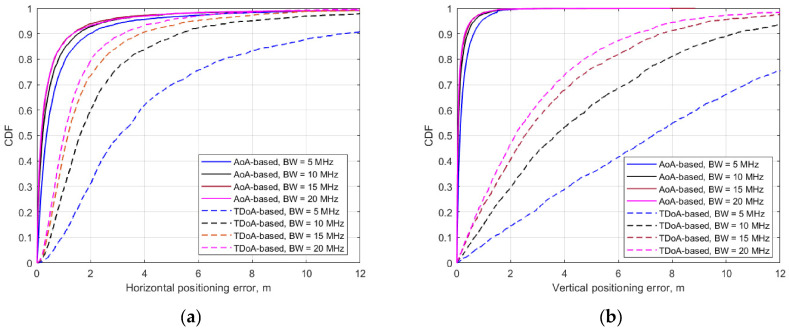
Localization performance comparison between the proposed (4, 4) URA AoA framework and a conventional TDoA baseline for the various system BWs (InF-SH, 30 kHz SCS): (**a**) CDF of horizontal positioning error; (**b**) CDF of vertical positioning error.

To further substantiate the benefit of the proposed AoA-based framework, its performance is benchmarked against a conventional TDoA localization framework under the identical InF-SH channel profile and network topology. The baseline TDoA system utilizes generalized cross-correlation with sub-sample peak interpolation to estimate relative propagation time delays, which are subsequently resolved into spatial coordinates via Chan’s closed-form hyperbolic localization algorithm. To ensure a conservative evaluation, benchmarking is performed using the minimum of the considered antenna configurations, i.e., the (4, 4) URA. Figure 10 presents this comparison for the various system BWs (InF-SH, 30 kHz SCS).

As illustrated in Figure 10, even the minimum (4, 4) URA configuration significantly outperforms the timing-based TDoA alternative across all evaluated bandwidths. While the 20 MHz TDoA baseline achieves a reasonable 90th percentile horizontal error of approximately 3.2 m, its positioning accuracy collapses entirely when the bandwidth is reduced to 5 MHz, yielding an 11.3 m error at the 90th percentile. This severe performance drop stems from the physical resolution limits of narrowband signals in dense multipath regimes. At 5 MHz, multiple path replicas in the time domain smear together, preventing the receiver from resolving individual ray arrivals. In contrast, the (4, 4) URA leverages spatial-domain wavefront regression to isolate these paths, restricting the 90th percentile horizontal error to 1.98 m and 1.27 m for the 5 MHz and 20 MHz bandwidths, respectively.

The architectural superiority of the proposed framework is even more pronounced in the vertical axis, where the TDoA framework suffers from catastrophic accuracy degradation. This vulnerability is a direct consequence of severe Vertical Dilution of Precision (VDOP) inherent to the industrial deployment geometry. Because the 3GPP InF-SH environment dictates an elevated, coplanar anchor configuration mounted at a height of 8 m, there is a complete absence of anchor altitude diversity relative to the ground-level target. Consequently, the TDoA hyperboloids flatten along the vertical axis, causing even minor multipath-induced timing fluctuations to map into massive vertical positioning ambiguities. Conversely, the proposed method bypasses this bottleneck by directly measuring the wavefront elevation angles. Even with a compact 16-element aperture, the spatial processing caps the 90th percentile vertical errors at 0.64 m and 0.28 m for the 5 MHz and 20 MHz bandwidths, respectively. This fully substantiates the selection of an AoA approach for coplanar industrial anchor deployments.

To provide insight into the algorithmic features enabling this superior spatial performance, the underlying metric behavior is evaluated.

The transition from a purely geometric approach to a quality-aware estimator is justified by the correlation analysis. The physical reliability of the proposed framework is statistically validated by the strong Spearman rank correlation between the plane-specific metrics defined in (34) and (35) and their respective angular errors. For the (8, 8) URA configuration at 5 MHz bandwidth (15 kHz SCS), the framework achieves correlation coefficients of 0.83 and 0.76 for the horizontal and vertical domains, respectively. These high correlation values confirm that the proposed metric serves as a dependable indicator of link quality, enabling the WLS estimator to effectively mitigate the impact of multipath-distorted measurements.

**Figure 11:**
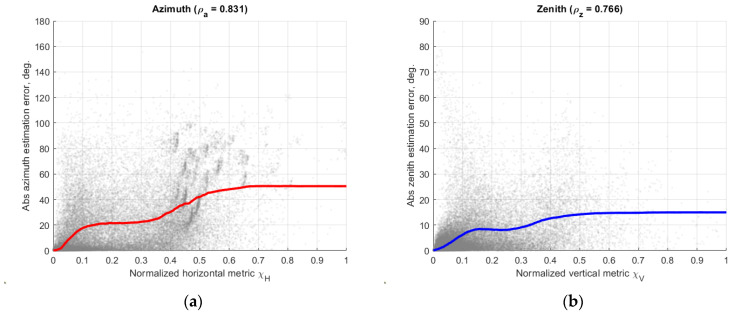
Correlation analysis for the (8, 8) URA (5 MHz/15 kHz SCS): (**a**) azimuth estimation error vs. normalized χ*_H_*; (**b**) zenith estimation error vs. normalized χ*_V_*. The point cloud shows the angle estimation error for different UE locations and channel realizations; the solid line denotes the sliding mean tracking the underlying trend.

The correlation analysis is illustrated in Figure 11 for the (8, 8) URA configuration using the minimum 5 MHz system bandwidth. The 15 kHz SCS configuration is selected for this characterization as it represents a critical RedCap deployment scenario. With a snapshot count of *L* = 288, it provides a statistically significant basis for mapping the relationship between phase residuals and estimation error.

To characterize the continuous relationship between wavefront distortion and estimation accuracy, a sliding mean (represented by the solid line) is superimposed on the scatter plots in Figure 11. This trend analysis reveals three distinct operational regimes: a linear predictive region for low distortion, a transition zone characterized by localized fluctuations, and a final saturation plateau. The smoothness of the sliding trendline highlights the deterministic nature of the proposed metric, confirming its utility as a robust weighting factor that enables the WLS estimator to effectively prioritize reliable measurements and maintain system-level stability.

## 4. Conclusions

5G indoor deployments are characterized by dense multipath conditions that hinder conventional time-domain positioning methods, such as TDoA, particularly for bandwidth-limited RedCap UEs. In such scenarios, narrow bandwidth prevents the resolution of the direct path, leading to significant performance degradation. This paper proposed a comprehensive uplink AoA-based positioning framework that shifts the computational burden to the network infrastructure, allowing for high-precision 3D localization while maintaining a single-antenna UE configuration.

The novelty of the proposed framework is centered on its unified estimation and weighting architecture, which treats AoA estimation and link-reliability assessment as an interconnected process rather than isolated tasks. The core of the framework is an intrinsic link quality metric generated as a direct, zero-overhead byproduct during the linear phase regression over the principal eigenvector. Statistical analysis confirmed a strong Spearman correlation between this metric and the actual estimation error, validating its use as a physically grounded proxy for angular uncertainty within a 3D projective geometry WLS estimator.

By bridging physical-layer wavefront planarity with a system-level positioning geometry, this architecture is designed to impart a critical “self-healing” capability to the positioning network. Instead of utilizing rigid, hard angular rejection thresholds, the framework applies an autonomous soft-filtering mechanism. This approach inherently suppresses heavily distorted multipath LoS or NLoS outliers within the matrix weight allocation itself, preserving the underlying geometric configuration of the TRPs.

Simulation results in a challenging 3GPP TR 38.901 InF-SH environment demonstrate that the framework comfortably achieves sub-meter horizontal and vertical accuracy even at the minimum 5 MHz bandwidth allocation, providing a robust and bandwidth-efficient alternative to ranging-based methods. This work demonstrates that existing communication antenna arrays can be leveraged for high-accuracy secondary services. By incorporating an uncertainty-aware weighting mechanism, the framework presents a self-correcting architecture that potentially assists in the mitigation of hardware impairments common in industrial settings.

While the proposed 3D AoA positioning framework demonstrates high efficiency and performance within the reference 3GPP InF-SH scenario, the current evaluation remains strictly simulation-based. Consequently, claims regarding practical robustness and immediate real-world deployment readiness must be interpreted within these boundary conditions. Acknowledging the lack of experimental hardware-level validation and limited scenario diversity as current limitations, future work will focus on validating the framework across broader hardware architectures and diverse industrial deployments.

Moving forward, the obtained UE coordinates can serve as a favorable initial estimate for high-precision carrier-phase positioning, where a coarse localization layer can facilitate resolving integer phase ambiguities. Furthermore, the framework can be extended to other bandwidth-limited network entities, such as Ambient IoT (A-IoT) tags, where hardware simplicity and computational efficiency are critical constraints.

## Figures and Tables

**Table 1:** Qualitative comparison of the proposed framework with common AoA baselines.

Feature	Bartlett	Capon/MUSIC	Proposed Framework
Estimation method	Search-based (beamforming)	Search-based (subspace)	Direct (principal eigenvector)
Complexity	High (2D grid search)	Very High (2D grid search) Moderate (root search)	Low
Narrowband robustness	High	Low/Moderate	High
Hardware Robustness	High	Low	High
Primary objective	Dominant path estimation	Multipath resolution	System-level robustness
System integration	Open-loop (estimator only)	Open-loop (estimator only)	Closed-loop (quality-aware)

**Table 2:** Simulation parameters.

Parameter	Value/Configuration
Channel Model Scenario	3GPP TR 38.901 Indoor Factory (InF-SH)
Multipath Modeling	Clustered Delay Line (CDL-C for NLoS/CDL-D for LoS)
Cluster/Ray Configuration	18–20 clusters; 20 sub-rays per cluster
Path Loss Model	3GPP InF Path Loss
Hall Dimensions	300 × 150 × 25 m
TRP Layout	3 × 6 Grid (50 m spacing)
TRP Height/UE Height	8 m/1.5 m
UE Velocity	3 km/h (Uniform Random Direction)
System Bandwidth	5 MHz/10 MHz/15 MHz/20 MHz
Subcarrier Spacing	15 kHz/30 kHz
SRS Allocation	Comb-2/2 consecutive OFDM symbols
TRP Antenna Configurations	(4, 4): 16-element URA, *d_H_*_λ_ = *d_V_*_λ_ = 0.5
	(8, 4): 32-element URA, *d_H_*_λ_ = *d_V_*_λ_ = 0.5
	(8, 8): 64-element URA, *d_H_*_λ_ = *d_V_*_λ_ = 0.5
UE Antenna Configuration	Single-antenna RedCap UE (Isotropic)
UE Transmit Power	23 dBm (Class 3 RedCap UE)
Noise Figure (TRP side)	5 dB

**Table 3:** Number of pilot subcarriers (effective bandwidth) for various SRS configurations.

Subcarrier Spacing	System Bandwidth			
	5 MHz	10 MHz	15 MHz	20 MHz
15 kHz	288	624	864	1152
	(4.32 MHz)	(9.36 MHz)	(12.96 MHz)	(17.28 MHz)
30 kHz	96	288	432	576
	(2.88 MHz)	(8.64 MHz)	(12.96 MHz)	(17.28 MHz)

## Data Availability

The data presented in this study are available within the article.

## Abbreviations

The following abbreviations are used in this manuscript:

AoA	Angle of Arrival
A-IoT	Ambient Internet of Things
BW	Bandwidth
CP	Carrier Phase
GDOP	Geometric Dilution of Precision
eMBB	Enhanced Mobile Broadband
IIoT	Industrial Internet of Things
LMF	Location Management Function
LoS	Line of Sight
ML	Maximum Likelihood
mMTC	Massive Machine-Type Communications
NLoS	None-Line-of-Sight
OFDM	Orthogonal Frequency-Division Multiplexing
OLS	Ordinary Least Squares
RedCap	Reduced Capability
RMS	Root Mean Square
RSS	Received Signal Strength
SRS	Sounding Reference Signal
TDoA	Time Difference of Arrival
ToA	Time of Arrival
TRP	Transmission and Reception Point
URLLC	Ultra-Reliable Low Latency Communications
UE	User Equipment
UL	Uplink
ULA	Uniform Linear Array
URA	Uniform Rectangular Array
WLS	Weighted Least Squares
